# A novel algorithm for maximum power point tracking using computer vision (CVMPPT)

**DOI:** 10.1371/journal.pone.0301363

**Published:** 2024-04-11

**Authors:** Morteza Ahmadi, Masoud Abrari, Majid Ghanaatshoar, Ali Khalafi

**Affiliations:** 1 Laser and Plasma Research Institute, Shahid Beheshti University, Tehran, Iran; 2 Centre for Quantum Technologies, National University of Singapore, Singapore, Singapore; 3 Department of Electrical Engineering, Iran University of Science & Technology, Narmak, Tehran, Iran; Vardhaman College of Engineering, INDIA

## Abstract

The behavior of an illuminated solar module can be characterized by its power-voltage curve. Tracking the peak of this curve is essential to harvest the maximum power by the module. The position of the peak varies with temperature and irradiance and needs to be traced. Under partial shading conditions, the number of peaks increases and makes it more difficult to find the global maximum power point (MPP). Various methods are used for maximum power point tracking (MPPT) that are based on iterations. These methods are time-consuming and fail to work satisfactorily under rapidly changing environmental conditions. In this paper, a novel algorithm is proposed that for the first time, utilizes computer vision to find the global maximum power point. This algorithm, which is implemented in Matlab/Simulink, is free of voltage iterations and gives the real-time data for the maximum power point. The proposed algorithm increases the speed and the reliability of the MPP tracking via replacing analogue electronics calculations by digital means. The validity of the algorithm is experimentally verified.

## Introduction

As the world’s energy demand is increasing, it is necessary to move toward renewable energy resources. Solar energy is considered as a resource that will provide much of the human energy in future. Solar cells that directly convert solar energy into electricity will have a key role as a future energy supply [1–3]. Usually, a single solar cell does not generate a huge amount of power, so a number of solar cells are connected in series to form a string with higher voltage and then the strings are connected in parallel to achieve a higher current and thus a higher power [4]. If any of these solar cells are shaded, they will work in reversed biased mode and instead of generating power, the cells under shading will dissipate the power produced by other cells. This condition, which is referred as partial shading, increases the power loss dramatically and can result in other serious problems including hotspots [5, 6]. In order to avoid the problems caused by partial shading, by-pass diodes are used in parallel with solar cells that create a current path to by-pass the shaded cells [7–9]. Using a single diode for every cell is not economical and so, usually, a diode is used for several solar cells. Therefore, various configurations such as series-parallel (SP), Bridge-linked (BL), Honey-comp (HC), and total-cross-tied (TCT) exist [10–13].

Adding by-pass diodes increases the non-linearity of a photovoltaic system and generates multiple peaks in its P-V curve [14–16]. To extract the maximum power of a photovoltaic system it is necessary for it to work in the global maxima [17]. Finding the maximum power point (MPP) under varying environment is a challenging task and several techniques are proposed to do so. One of the techniques which are used to find the global maximum is perturb and observe (P&O) method that uses perturbation steps to find the MPP [18–20]. In this algorithm, which is also called the hill climbing algorithm, the operating point is never steady at the MPP but is drifting around it. Another problem with this method is that in the condition of rapidly changing environmental lighting, the algorithm fails in its convergence effort. Another method which is used for MPP tracking is incremental conductance algorithm that solves the problems of the P&O algorithm by means of smaller sampling intervals but increases the system complexity and makes its hardware implementation harder [21]. A huge amount of studies has been conducted to improve MPPT methods that have resulted in adaptive perturb and observe method and variable step size incremental conductance method [22–24]. Another method for MPPT is Constant Voltage (CV) [25]. The MPP is considered to have a set voltage value in the CV technique, which is the same as the value recorded under the standard test conditions (STCs) specified by the manufacturer. Usually, this constant voltage falls between 72% and 80% of the V_OC_. A feedback control loop is then utilized to modify the MPPT converter’s duty cycle using this reference voltage. All that is needed to build a constant voltage MPPT control is measuring the array voltage. It can be put into practice using both digital and analog electronics. Ripple Correlation Control (RCC) is another technique which is based on the ripples in the voltage and current [26]. There are ripples in the voltage and current when a PV array is linked to a power converter. The switching operation of the converter causes these waves to be applied to the PV array. Ripples therefore also have an impact on the PV array’s power. RCC is a tracking method that tracks the MPP by monitoring ripples in voltage and current. The derivatives of the changing PV power are correlated with those of the fluctuating PV voltage or current in order to attain the MPP and push the power gradient to zero. When power grows while current or voltage increases, the operating point is below the MPP. On the other hand, the operating point climbs above the MPP if the power is cut off and the current or voltage increases. Open Circuit Voltage (OCV) technique is also a way to track the maximum power point [27]. According to the OCV technique, the voltage at MPP is determined by multiplying the solar module’s V_OC_ by a constant coefficient that falls between 0.7 and 0.8. Because of its streamlined process, this approach is simple to use, but it requires disconnecting the load each time the V_OC_ needs to be measured, which reduces system efficiency and causes power supply interruptions. As a result, it is not advised to apply this technique in situations when the load’s supply continuity is crucial. Short Circuit Current (SCC) is another method for MPPT [28]. This tracking technique is predicated on the observed linear relationship between the short-circuit current and the PV current at MPP, much like the OCV technique. SCC uses a proportional constant, which is mostly influenced by the filling factor, weather conditions, and PV cell technology. The constant for polycrystalline photovoltaic modules is estimated to be around 0.85. Many times, a PV scan is performed every few minutes to determine the constant. Once acquired, the system employs the revised approximation until the subsequent computation is executed. After then, the control flowchart and the OCV approach are similar. Consequently, this approach shares the same advantages and disadvantages as the OCV control method. All of the mentioned methods have an iterative nature that is time-consuming and may face problems in rapidly changing conditions. For such conditions, other algorithms like distributed MPPT, PV curve scan, and other algorithms are proposed which have a high level of complexity and in some cases, they require to change the hardware configuration based on shading condition [29–33]. There are other algorithms, among which someone can refer to Low Burden Narrow Search (LBNS) [34], butterfly optimization algorithm [35] and fuzzy logic control (FLC) MPPT [36]. For a complete review of the MPPT methods, one can see [37].

Computer vision is a science that is concerned with the automatic extraction, analysis, and understanding of useful information from a single image or a sequence of images [38]. By this means, one can detect the modules and shadings on them, useing the data to simulate the partial shading condition and determine the maximum power point. In many studies, the simulation data has been well matched to the experimental results [39, 40]. Furthermore, it has been confirmed that the performance of the solar modules is mostly affected by the shading pattern rather than its strength and volume [41]. Hence, it is possible to evaluate the performance of the solar modules by detecting the shading patterns on them. Computer vision techniques have been previously used for solar cell crack inspection [42], fault diagnosis [43] and shading detection [44]. However, there is no report of maximum power point tracking under partial shading conditions using computer vision yet.

In this paper, a new approach for MPP tracking (CVMPPT) is proposed. The basic idea behind our algorithm is that in contrast to all the previous MPPT methods that utilize analogue electronics, one can use computer vision to detect shading patterns on solar cells and use the acquired data to calculate the voltage at maximum power by means of mathematical modeling. This method has no voltage iterations and finds the global maximum power point in a single step. it is unlikely to fail in its MPP tracking effort due to its reliance on mathematical analyses. The proposed algorithm is implemented in Matlab/Simulink and verified experimentally. The paper is organized as follows; in section 2, the solar module model and its parameters are described, in section 3 the flowchart of the algorithm is proposed and explained, while the CVMPPT algorithm is implemented and experimentally verified in section 4.

### Solar cell model & simulation

**Fig 1:**
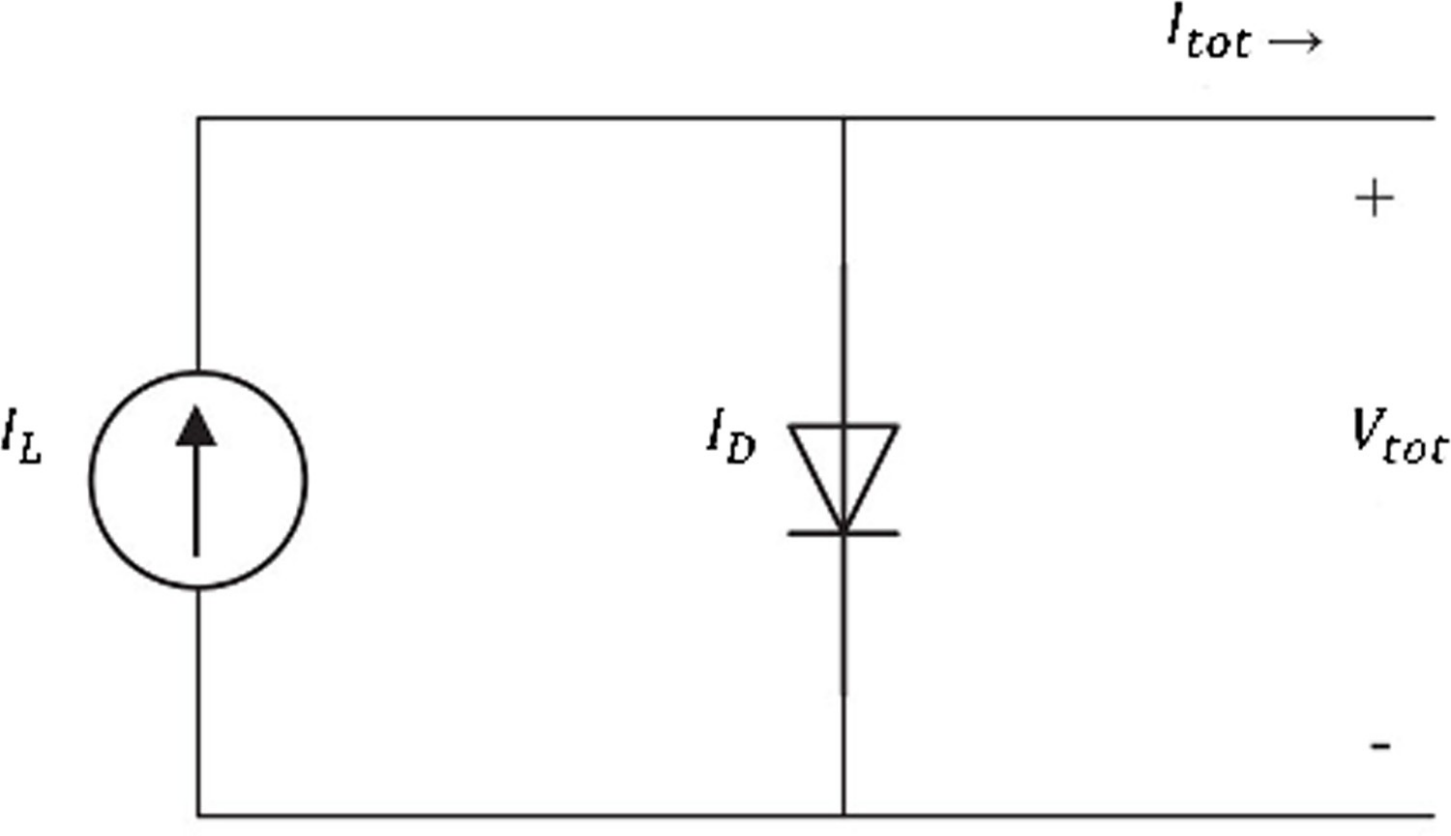
The equivalent circuit for a solar cell.

Equivalent circuits are used to describe the behavior of solar cells [45]. These circuits are formed by a current source, one or two diodes, and shunt and series resistances. The same circuits can be employed for solar modules and arrays [46]. Fig 1 shows the single diode solar cell mode, in which a diode and a current source are connected in parallel.

**Fig 2:**
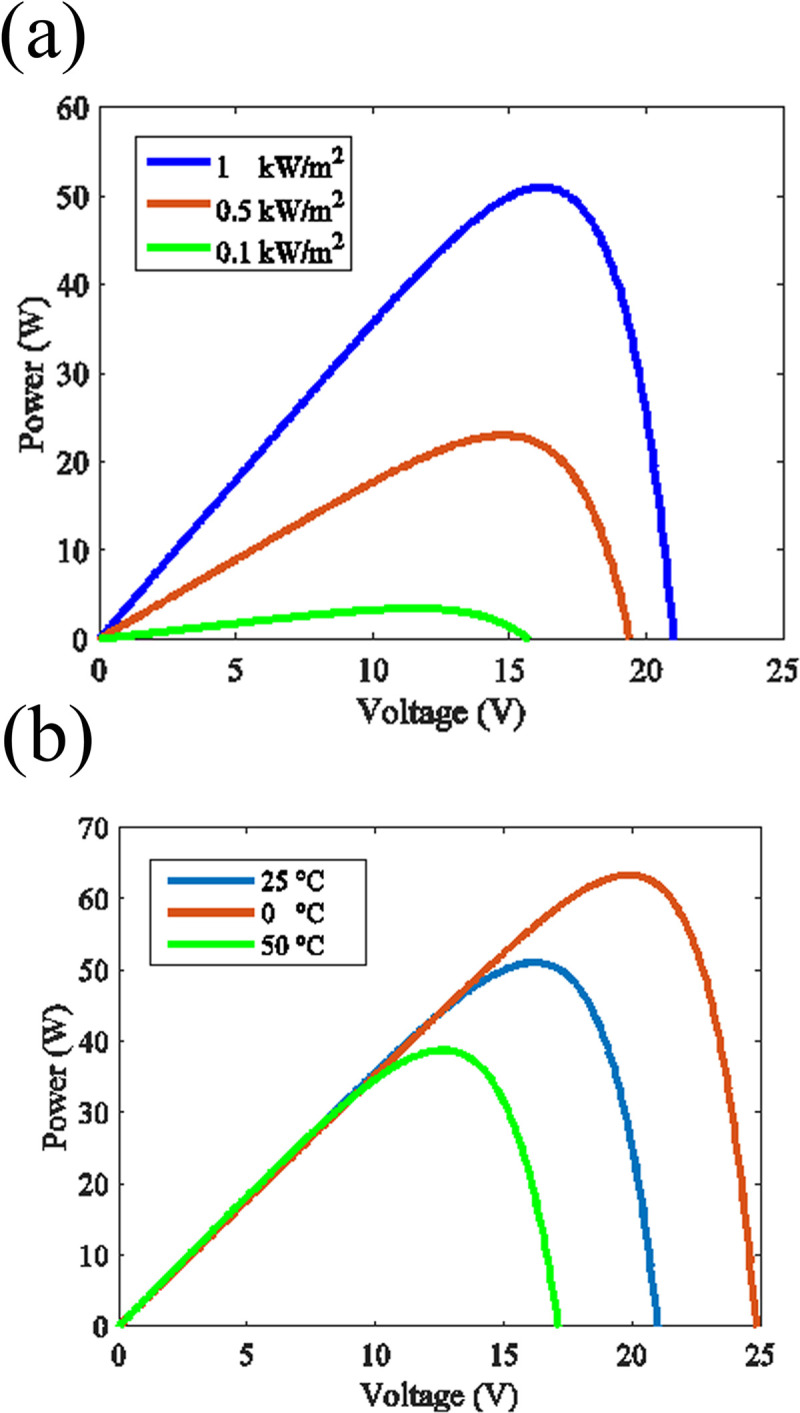
**a.** The influence of irradiance on P-V curve of a solar module at a fixed temperature of 25°C, **b.** variation of a solar module P-V curve with temperature at an irradiance of 1 kW/m^2^.

The acquired current of a solar cell can be expressed as:

$$I_{tot}=I_{L}-I_{D}=I_{L}-I_{0}\left[\exp\left(\frac{qV_{tot}}{nk_{B}T}\right)-1\right]$$ (1)

where *I*_*L*_ is the photo-generated current, *I*_*D*_ is the diode current, *I*_0_ is the diode reverse saturation current, *q* stands for the elementary charge, *V*_*tot*_ is the voltage across the diode, *n* represents the diode ideality factor, *k*_*B*_ is the Boltzmann’s constant and *T* is the temperature of the device. As can be seen from Eq 1, the performance of a photovoltaic system depends on the temperature and the amount of incident irradiance. Typically, solar cell behavior is demonstrated by plotting its voltage against its current, a graph named as I-V curve [47]. For MPP tracking purposes it is more convenient to use P-V curve which is simply acquired by multiplying the current at each voltage by its respective voltage [48]. The effect of temperature and irradiance on the P-V curve of a solar module is illustrated in Fig 2 which is acquired by solving Eq 1 using Matlab/Simulink. It can be seen from Fig 2 that reducing the amount of irradiance on the module dramatically decreases the generated power. This figure shows that how shading can affect a single solar module behavior and consequently disturb a solar array performance.

**Fig 3:**
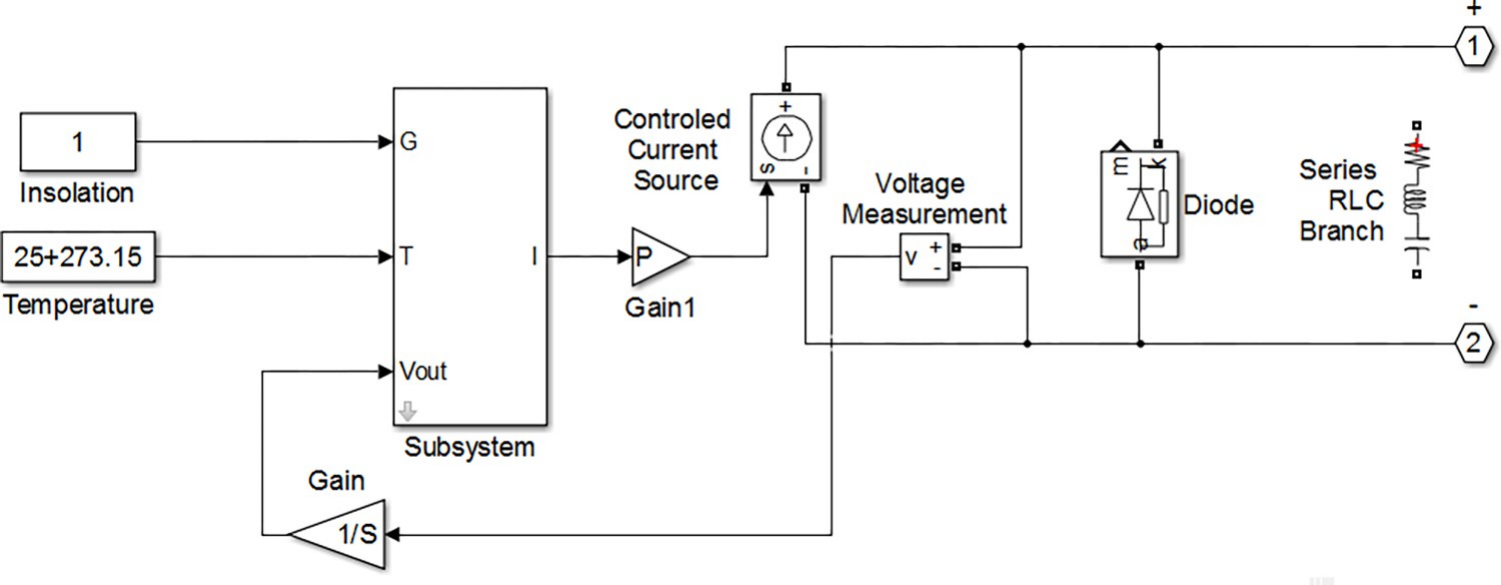
The employed solar module in Matlab/Simulink.

Fig 3 shows the implementation of Eq 1 in Matlab/Simulink. This is the same circuit proposed in our previous work [41]. In this circuit, the properties of the solar cell such as V_OC_ and I_SC_ are defined in the subsystem, which solves Eq 1 by acquiring the amount of insolation and temperature as input and gives us the values of the module output voltage and current. Partial shading situations can be examined by reducing the insolation amount on specific modules in an array. The output terminals of this model can be connected to other cells, which enables us to simulate various configurations that are common in photovoltaic systems. In this paper, SP configuration has been used to implement the algorithm due to its popularity among other configurations.

### Description of the algorithm (CVMPPT)

**Fig 4:**
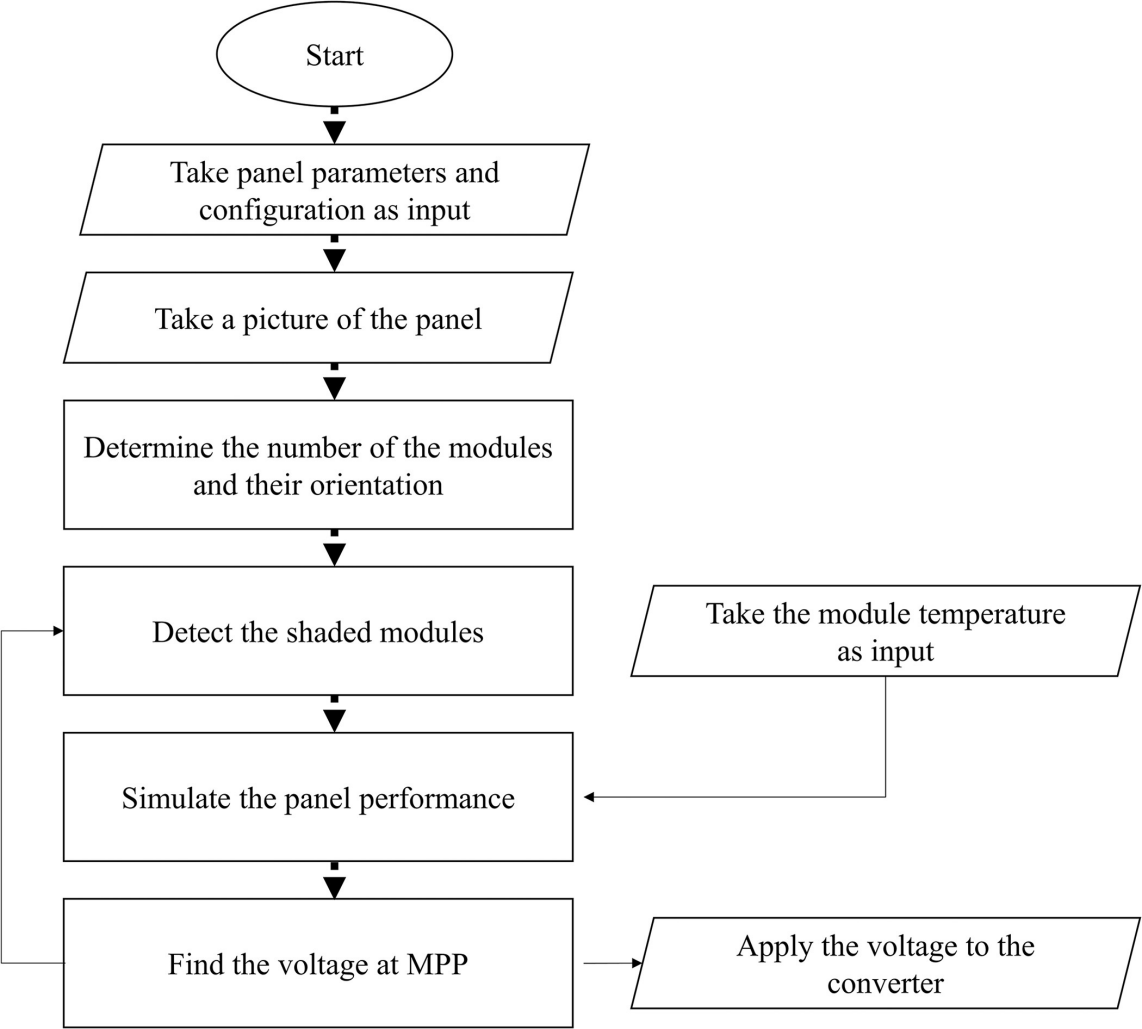
Flowchart of the CVMPPT algorithm.

Here an algorithm is proposed, which takes a completely new approach for MPP tracking. The complete flowchart of the algorithm is illustrated in Fig 4. The description of the algorithm is as follows.

#### Step 1

At the beginning, the panel must be defined for the computer. Several parameters such as open circuit voltage, short circuit current, the ideality factor and other parameters should be considered. If a commercial solar device is employed, the required information can be extracted from their datasheets. In many cases, there are digital libraries containing the needed information. In this step, the configuration of the solar panel (e.g. SP, TCT, BL & HC) should also be provided by the user. Computer vision can only extract the visual data and has no information of the back contacts and interconnections of the solar cells, which can play a determinant role.

#### Step 2

**Fig 5:**
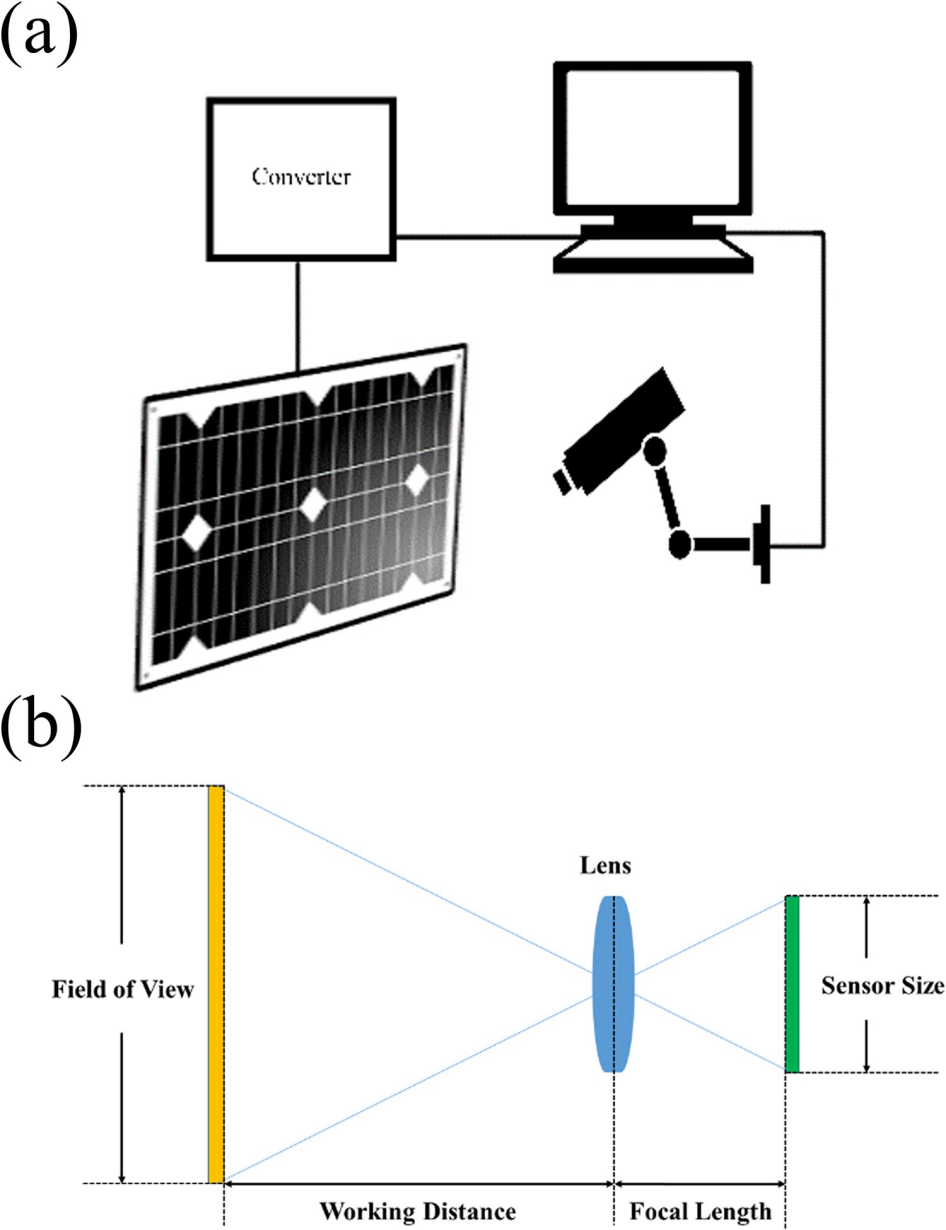
**a.** Schematic of the algorithm implementation, **b.** The camera parameters.

A picture of the defined panel is taken in this step. This can be done by simply placing a camera in front of the solar panel or panels as it is illustrated in Fig 5A. The camera should be placed in a way that it does not cause any shading on the panel. The minimum resolution of the camera is determined by several parameters [49]; the field of view (FOV) which is the area covered by the solar panel(s), the working distance (WD) that the camera is installed, and the smallest feature which in our case is the area of single module in the panel. Additional parameters that play a role in camera selection are its pixel size and focal length. Fig 5B illustrates the geometry, which the camera is placed in front of the solar panel(s). In this figure, the camera is defined by 3 parameters, namely its sensor size which is the number of pixels in each row and column of the camera’s CCD or CMOS sensor, its focal length and its lens. If the algorithm is used for a large-scale solar plant, either a high-resolution camera, which covers all the panels, or a cheap low-resolution camera for each cluster of the panels can be used.

The resolution of an image is the number of pixels in the image. To make an accurate measurement on the image, a minimum of four pixels per smallest feature is needed. According to the Nyquist–Shannon theorem, two samples of a continuous signal are required to define it as a discrete signal, which contains all the information. Since the images of the solar panels are 2D signals, it is better to use a minimum of 4 points to detect the smallest feature [50]. Then, to calculate the minimum sensor resolution, the size (in real-world units) of the field of view is divided by the size of the smallest feature and multiply by four as:

$$Camera\;resolution=4(\frac{Field\;of\;view(FOV)}{Smallest\;feature}).$$ (2)

In order to define the distance of the camera to the panel(s), the camera properties must be taken into account. The working distance of the camera is calculated by:

$$Working\;distance=\frac{Focal\;lenght\times FOV}{Sensor\;size}.$$ (3)

The proposed algorithm can be adjusted for various solar panel topologies. If solar arrays are spread in far distances from each other, it is suggested to use a single CVMPP tracker for each array. on the other hand, if the solar arrays are clustered together, using a single CVMPP tracker for all of them can reduce the system price. In any case, an unshaded image of the panels is needed to start the algorithm.

#### Step 3

The image is processed to determine module positions in the image. Solar modules usually have a distinct pattern and are in specific shapes such as square or octagonal. This makes it easier to find the module positions in the image. There are many pattern recognition and shape finding algorithms in computer vision libraries such as OpenCV [51]. The solar panels which are used in this paper have rectangular modules, so built-in Matlab functions is used for determining rectangles in the image to extract the module position data. Once the modules are detected in the image, they must be assigned to the previously defined panels in step 1. This process is only carried out once and is not needed until the panel’s structures are changed. The assignment is done manually but it can also be performed by artificial intelligence techniques.

#### Step 4

The status of the solar panel (either partially shaded or unshaded) must be clarified at this step. For this purpose, successive images are taken from the modules. If a module is shaded, the subtraction of two consecutive images of that module would be non-zero. By this means, one can detect the shaded modules and also the shading strength. Of course, this is not a reliable method for shading detection because even in the unshaded situations, the subtraction of images is not zero due to the changes in lighting during the day. Thus, a threshold for the images difference is set. If the subtraction of images is bigger than the threshold, then it is assumed as the shading and if not, it is due to the lighting situation. The threshold is a function of insolation during the day which itself depends on the geographical location of the device and can be set by trial and error.

#### Step 5

At this step, the performance of module, either shaded or unshaded, is simulated. There are many models that can be used for simulation of solar cells under partial shading condition. In this paper, a model implemented in Matlab/Simulink have been used to illustrate the feasibility of the algorithm. Matlab/Simulink is a high-level programming tool that needs certain hardware requirements. If the algorithm is written in a lower-level programming language, it will have a much higher speed and can also be implemented in affordable hardware such as microcontrollers. The output of this step is the I-V and P-V curves of the solar panel.

#### Step 6

By having the characteristics curves of the solar panel, the MPP can be easily detected. The voltage at MPP is given to the converter to apply it on the solar panel. After this step, the algorithm returns to the fourth step and repeats the procedure. Since the configuration of the solar modules is not changed, the previous steps are not necessary for the routine but since the solar cell parameters are degraded over time, a calibration step must be performed in a timely basis to ensure the MPP tracking precision and persistence.

In this algorithm, the MPP is detected and applied by means of computer vision technique. There are several image processing methods that can be used to detect the modules positions and shading on them, namely the ant colony optimization (ACO) [52], the edge detection method [53], binarization [54], histogram model [55], statistical model [55], artificial neural networks (ANNs) [56], thermal image processing [57], color matching and texture matching algorithms [58]. In order to detect the modules in this work, the “regionprops” Matlab function with ’BoundingBox’ argument is used, which detects rectangular objects in the image using connected components detection. The detected rectangles are optimized to only match the solar modules. After the modules positions are spotted, Matlab “rectangle” function is used to show the position of the detected module.

### Implementation of the algorithm

The solar panel installed on the rooftop of the faculty of electrical engineering at Shahid Beheshti University was used for image processing. The solar panel had poly-crystalline silicon modules with a maximum power of 50 W_p_ and open circuit voltage of 21 V. The panel consisted of 2×4 modules, which their terminals could be connected in any desired configurations. The series-parallel configuration is chosen in this work. A thermometer can be used to measure the solar panel temperature and a single solar cell is used to measure the irradiance value. In this simulation, we have considered the temperature of the solar panels as 22°C and the irradiance as 900 W/m^2^. A Canon EOS 500D camera is used to take the modules pictures.

**Fig 6:**
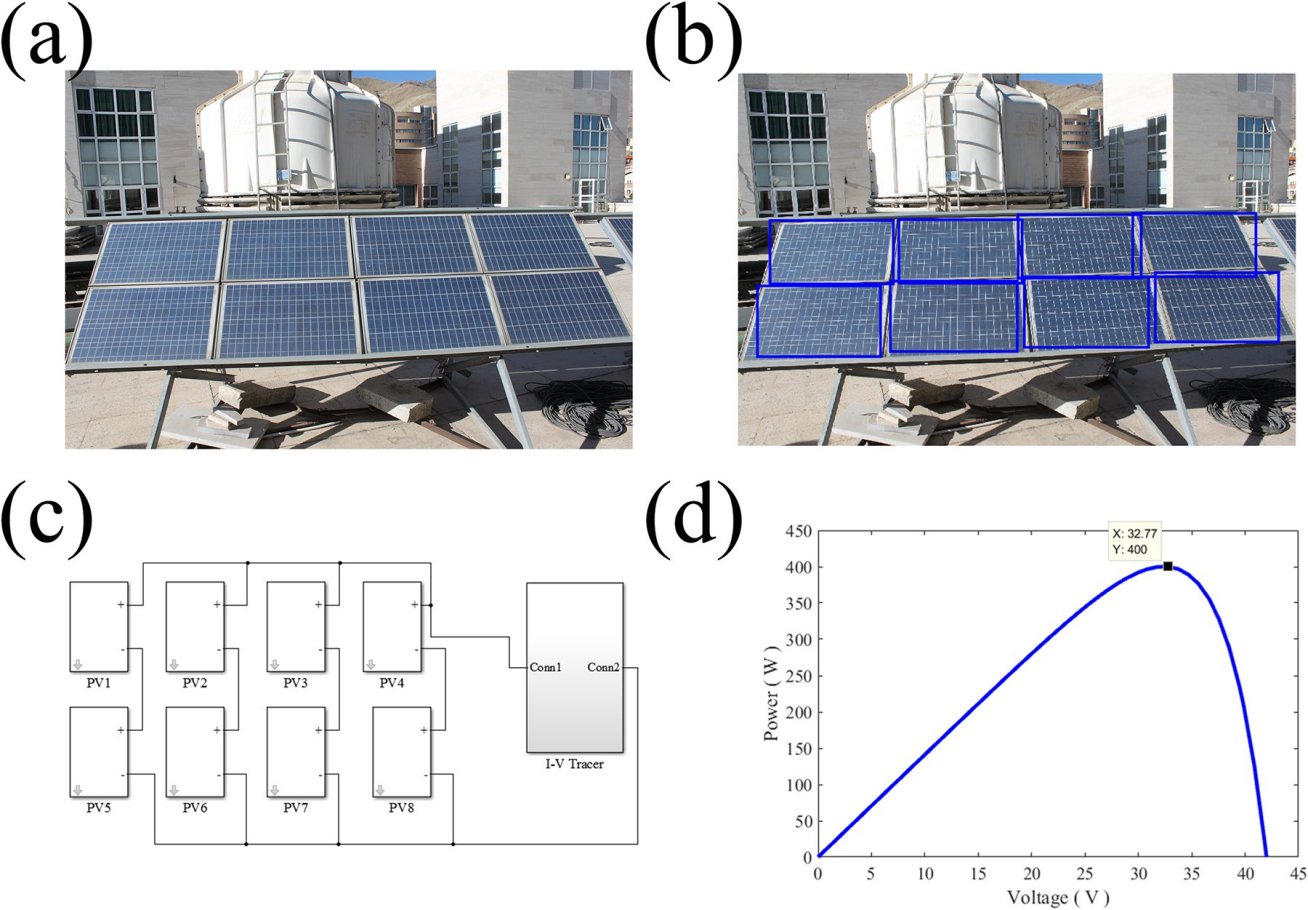
**a.** The solar panel used for shading detection, **b.** The detected solar module positions in the image, **c.** Matlab/Simulink implementation of the solar array and **d.** the calculated P-V curve and MPP.

Fig 6A shows the image of the solar panel used for shading detection. As can be seen from the image, the modules are in 2×4 configuration and have a rectangular shape. This rectangular shape makes it easier to detect the module positions by computer vision. The detected modules of the solar panel are shown in Fig 6B. When the modules are detected, the panel configuration must be defined in Matlab/Simulink environment. After the partially shaded module is detected, its coordinates in the solar array are communicated to the Simulink and Simulink considers the module under partial shading which can be simulated and the MPPT can be estimated using simulation. The 2×4 implementation of the solar panel using the solar modules shown in Fig 3. with SP configuration is illustrated in Fig 6C. Each module in Matlab/Simulink environment should be assigned to its corresponding real module. This job is only performed once and is not needed until the solar panel is changed. Each module position is manually assigned in the image to its corresponding real module. By having the solar module’s data and its configuration the P-V curve of the solar panel can be calculated and its maximum power point is determined through mathematical analysis by Matlab. The P-V curve of the solar panel and its MPP acquired by Matlab is demonstrated in Fig 6D. The maximum power point was detected at 32.7 V with a power of around 400 W.

The partial shading on a single module is shown by the change in the irradiance of that module which manifest itself as the decrease in the current produced by the cell. This drop in the current acts similar to a cell under partial shading condition in practical solar cells. In Simulink, this situation is simulated by making arrays of individual solar modules under partial shading, full shading or no shading conditions and track the maximum power point in the full array. In the case of shading occurrence, the shading pattern on the panel is detected by the computer and is applied to the Simulink model, the P-V curve of the new situation is calculated and the new MPP can be acquired. In this situation, the number of power peaks increases that makes MPP tracking more complicated for other methods such as P&O or incremental conductance. But since our proposed algorithm takes a numerical approach for tracking the global maximum point, it does not sense any problem in finding the MPP in comparison to the unshaded condition. This is one of the main advantages of CVMPPT algorithm over previous methods. The proposed algorithm procedure is not affected by the shading pattern and can track the MPP under any shading circumstances. All of these steps are performed real-time and the maximum power point can be acquired at any given time. Unlike previous MPP tracking methods, voltage iterations are not witnessed in the proposed algorithm and the only iteration is in the processor which solves Eq 1 numerically for each module and takes the effect of shading into account on the P-V curve of the panel. The algorithm implementation is quite straightforward and does not require complex hardware.

**Fig 7:**
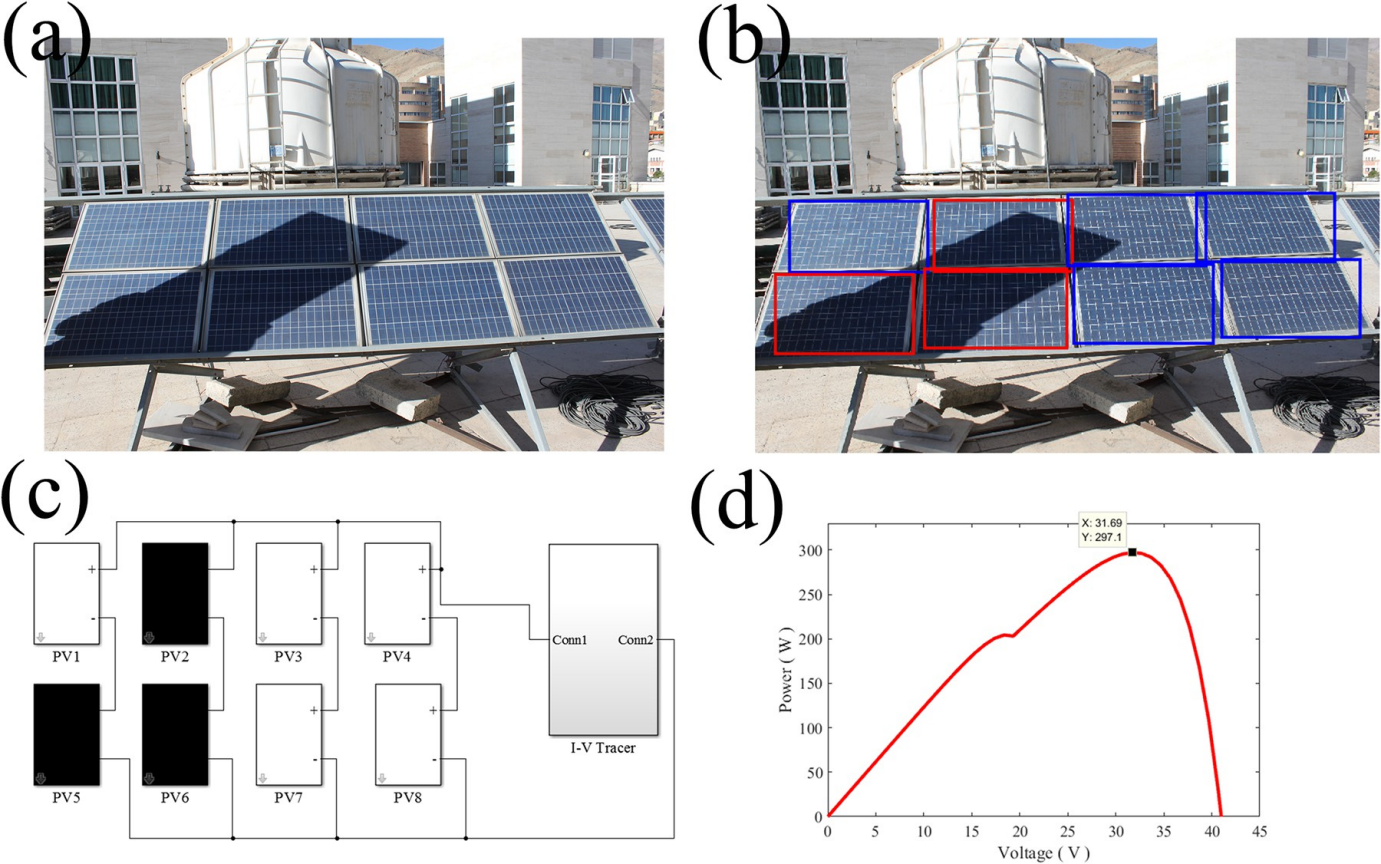
**a.** Intentional shading, **b.** detected shading pattern, **c.** Matlab/Simulink implementation of the solar array under shading and **d.** the calculated P-V curve and the MPP under shading.

In this study, the solar panel that is shown in Fig 7A is deliberately shadowed. The shading pattern is successfully detected by computer vision (Fig 7B and analyzed in Matlab/Simulink (Fig 7C). For our employed solar panel, after the intentional shading occurrence, the maximum power is dropped to 297.1 W at the voltage of 31.7 V, which is correctly traced by our proposed algorithm (Fig 7D).

### Experimental verification

**Fig 8:**
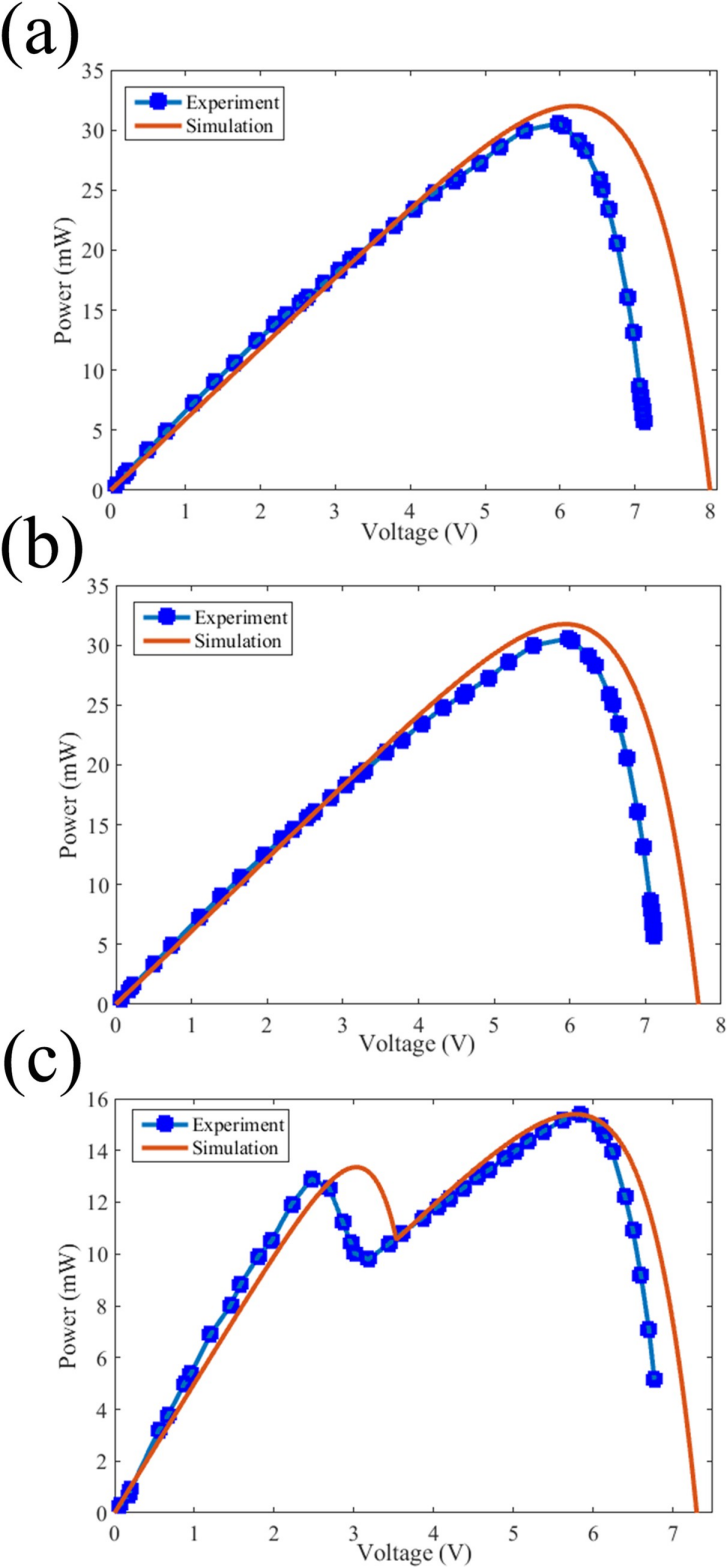
The experimental and calculated data for **a.** unshaded condition before calibration, **b.** unshaded condition after calibration and **c.** shaded condition.

In order to verify the MPP tracking of the algorithm, 8 SBE9060 polycrystalline silicon solar modules with a V_OC_ of 4 V and efficiency of 16% are put into SP configuration along with 1N4007 MIC by-pass diodes. The solar array is illuminated by a 12-watt white light LED. The current-voltage curve of the solar array configuration was acquired by point-to-point measurement of the output voltage and current using digital multimeters. The same circuit was implemented in Matlab/Simulink. The modules were intentionally shaded with different scenarios in order to simulate the partial shading condition. The measured experimental data and the calculated P-V of the unshaded array for one of the shading scenarios in which two neighboring cells are shaded is shown in Fig 8A. As it can be seen from the image, the experimental and analytical curves are not matched with each other. This is a typical difference that is usually witnessed between measurement and mathematical analysis. Therefore, a calibration step is required to be performed to fit the analytical data to the experiment before it can be used for MPP tracking. For this purpose, the following equations are simply used which fit the MPP voltage and power from the simulation to experiment:

$$V_{{Simulation}_{new}}=\frac{V_{MPP\_Experiment}}{V_{MPP\_Simulation}}\times V_{Simulation},$$ (4A)

$$P_{{Simulation}_{new}}=\frac{P_{MPP\_Experiment}}{P_{MPP\_Simulation}}\times P_{Simulation}.$$ (4B)

The P-V curve of the solar array after calibration step is shown in Fig 8B. After this step, the MPP for any condition, either shaded or unshaded can be calculated using the proposed algorithm. The P-V curve of the partially shaded solar array along with the calculated curve is demonstrated in Fig 8C. The MPP of both curves is almost the same and so the algorithm can be used to trace the MPP of the solar arrays. The algorithm was tested for different shading scenarios. In any case, it could detect the MPP with reliable error of around 1%. The fact that the proposed algorithm utilizes digital processing instead of common MPP algorithms which are based on analogue power electronics provides a better reliability for the system [59]. It reduces the complexity of the electronics and transfers the processing step into the computer. Another advantage of the proposed algorithm is that the number of power perturbation steps does not depend on the location of the global peak, and it takes nearly the same time to track the MPP in each case while in SSJ-GMPPT and R-GMPPT the iteration steps change on global peak location [60, 61].

The quantitative comparison of different MPPT methods is presented in Table 1. In this table, the tracking accuracy, system complexity, and convergence speed of these methods are compared to CVMPPT. It is observed from the experimental results that the CVMPPT gives a more economic and quicker response under different shading scenarios. As it can be seen from the table, our proposed algorithm performs the MPP tracking in a single power perturbation step and will definitely act faster than the previous methods. The processor iteration steps in our method are significantly more than other methods, however, it must be kept in mind that a thousand iteration in a processor with a speed in the range of several megahertz is done in a matter of milliseconds which can be considered almost real-time. This is a much faster performance in comparison with the perturbation steps in power electronics which can take up to several seconds.

**Table 1:** Comparison of the different MPPT algorithms.

Algorithm	Power Perturbation steps (delay)	Processor iteration steps	Complexity	Accuracy (%)	Reference
PV curve scan	95	NA	low	97	[62]
DMPPT	75	NA	high	97	[63]
GBSMPPT	17	NA	low	98.3	[39]
CVMPPT	1	~10000	low	~99	This work

## Conclusion

The CVMPPT algorithm proposed in this paper uses a camera to detect the shading pattern on solar panels and the data is used to detect the MPP by solving fundamental equations of the solar cells. In contrast to all the previous maximum power point tracking methods which employ analogue electronics and voltage iterations to detect the MPP, the CVMPPT algorithm relies on image processing and mathematical analysis. This feature makes the CVMPPT algorithm superior to the previous methods in terms of reliability, speed and accuracy. The experimental verification of this algorithm confirmed the advantages of CVMPPT to the previous methods and thus we believe that the CVMPPT can replace current MPPTs in the large and small scale solar facilities.
